# MicroRNA-218 functions as a tumor suppressor in lung cancer by targeting IL-6/STAT3 and negatively correlates with poor prognosis

**DOI:** 10.1186/s12943-017-0710-z

**Published:** 2017-08-22

**Authors:** Yan Yang, Lili Ding, Qun Hu, Jia Xia, Junjie Sun, Xudong Wang, Hua Xiong, Deepak Gurbani, Lianbo Li, Yan Liu, Aiguo Liu

**Affiliations:** 1 Department of Pediatrics of Tongji Hospital, Tongji Medical College, Huazhong University of Science & Technology, Wuhan, 430030 People’s Republic of China; 2 Experimental Medicine Center of Tongji Hospital, Tongji Medical College, Huazhong University of Science & Technology, Wuhan, 430030 China; 3 Department of Medicine, Division of Epidemiology, Vanderbilt University, Nashville, TN 37232 USA; 4 Department of Biochemistry, UT Southwestern Medical Center, Dallas, TX 75390 USA; 5 Department of Oncology of Tongji Hospital, Tongji Medical College, Huazhong University of Science & Technology, Wuhan, 430030 China; 6 Department of Radiation Oncology, UT Southwestern Medical Center, Dallas, TX 75390 USA; 7 School of Biological Science and Technology, University of Jinan, Jinan, China

**Keywords:** microRNA-218, Lung cancer, STAT3, Interleukin-6 receptor, Cancer stem cell

## Abstract

**Background:**

Aberrant expression of microRNAs in different human cancer types has been widely reported. MiR-218 acts as a tumor suppressor in diverse human cancer types impacting regulation of multiple genes in oncogenic pathways. Here, we evaluated the expression and function of miR-218 in human lung cancer and ALDH positive lung cancer cells to understand the potential mechanisms responsible for disease pathology. Also, the association between its host genes and the target genes could be useful towards the better understanding of prognosis in clinical settings.

**Methods:**

Publicly-available data from The Cancer Genome Atlas (TCGA) was mined to compare the levels of miR-218 and its host gene *SLIT2/3* between lung cancer tissues and normal lung tissues. Transfection of miR-218 to investigate its function in lung cancer cells was done and in vivo effects were determined using miR-218 expressing lentiviruses. Aldefluor assay and Flow cytometry was used to quantify and enrich ALDH positive lung cancer cells. Levels of miR-218, IL-6R, JAK3 and phosphorylated STAT3 were compared in ALDH1A1 positive and ALDH1A1 negative cells. Overexpression of miR-218 in ALDH positive cells was carried to test the survival by tumorsphere culture. Finally, utilizing TCGA data we studied the association of target genes of miR-218 with the prognosis of lung cancer.

**Results:**

We observed that the expression of miR-218 was significantly down-regulated in lung cancer tissues compared to normal lung tissues. Overexpression of miR-218 decreased cell proliferation, invasion, colony formation, and tumor sphere formation in vitro and repressed tumor growth in vivo. We further found that miR-218 negatively regulated IL-6 receptor and JAK3 gene expression by directly targeting the 3′-UTR of their mRNAs. In addition, the levels of both miR-218 host genes and the components of IL-6/STAT3 pathway correlated with prognosis of lung cancer patients.

**Conclusions:**

MiR-218 acts as a tumor suppressor in lung cancer via IL-6/STAT3 signaling pathway regulation.

**Electronic supplementary material:**

The online version of this article (doi:10.1186/s12943-017-0710-z) contains supplementary material, which is available to authorized users.

## Background

In China, lung cancer is the most common incident cancer with high mortality rate [1].Tumor heterogeneity and poor prognosis are two major factors responsible for high mortality of lung cancer patients. In fact, among the two major lung cancer subtypes, non-small-cell lung carcinomas (NSCLC) weights more than 80% than small cell lung cancer (SCLC). The three main subgroups of NSCLC include adenocarcinoma, squamous cell carcinoma and large cell carcinoma. Adenocarcinoma is by far the more prominent type than squamous cell carcinoma [2]. Over the past several years, substantive progress has been made towards diagnosis and treatment strategies for lung cancer subtypes, but the overall 5-year survival rate of lung cancer is no more than 18% [3]. Therefore, a better understanding of the molecular mechanisms underlying the development and progression of lung cancer is needed to improve early disease diagnosis and also provide newer therapeutic strategies for better disease control.

MicroRNAs (miRNAs) are a class of single-stranded, non-coding RNAs of 19–25 nucleotides that serve as negative regulators of gene expression by interacting with 3’untranslated regions (3’UTRs) of the target genes [4].Aberrant expression of miRNAs has been reported in different diseases including lung cancer, where they may act as oncogenes or tumor suppressors [5]. MiR-17-92 [6], miR-21 [7], and miR-221/miR-222 [8] were reported to promote lung tumorigenesis, while let-7 [9], miR-126 [10], miR-16 [11], miR-340 [12], miR-145 [13], andmiR-373 [14] act as tumor suppressors.

MiR-218 is a vertebrate-specific intronic miRNA co-expressed with its host genes, tumor suppressor gene *SLIT2/3*. The mature form of miR-218 is generated from two separate loci, miR-218-1 and miR-218-2, which are located at the introns of *SLIT2* and *SLIT3*, respectively [15–17]. Several lines of evidence suggests that the miR-218 is depleted in some human solid cancers, such as cervical cancer [16], lung squamous cell carcinoma [17], bladder cancer [18], glioma [19], and gastric cancer [20], where tumor cell invasion and proliferation are relatively enhanced. However, the molecular mechanism of miR-218 in NSCLC remains unclear.

In this present study, we attempted to determine the publicly-available data from The Cancer Genome Atlas (TCGA) for comparison of miR-218 and its host gene *SLIT2/3* expression levels between lung cancer tissues and normal lung tissues. We also investigated the downstream targets of miR-218 in lung cancer cells for its underlying mechanism of action. Finally, we report the correlation between the levels of miR-218 host genes, as well as its targeted genes, and the prognosis of lung cancer disease.

## Methods

### Cell culture, transfection and infection

Human lung cancercell lines H1975 and A549 were purchased from the American Type Culture Collection (ATCC, Manassas, VA). Cells were cultured in RPMI 1640 medium (Gibco/Life Technologies, Grand Island, NY) supplemented with 10% fetal bovine serum (FBS) and 1% penicillin–streptomycin at 37 °C in a humidified atmosphere with 5% CO_2_. H1975 and A549 cells were transiently transfected with miR-218 mimic or miR-218 inhibitor or small interfering RNA (siRNA) (Ambion/Life Technologies, Grand Island, NY; Sigma-Aldrich, St. Louis, MO) using Lipofectamine RNAiMAX Reagent (Life Technologies, Grand Island, NY) as per manufacturer’s protocol. Co-transfection of the miRNA mimic and plasmid DNA was conducted using Lipofectamine 2000 Reagent (Life Technologies, Grand Island, NY). Lentivirus vector expressing miR-218 was purchased from Applied Biological Materials (Richmond, BC). Lentiviruses were prepared according to the manufacturer’s protocol.

### Invasion assay

Cell invasion assay was performed in 24-well transwell chambers(Corning, New York, NY) containing polycarbonate filters with 8 μm pores coated with matrigel (Corning, New York, NY).H1975 and A549 cells were transfected with miR-218 or miR-control. Forty-eight hours after transfection,1 × 10^5^ cells suspended in serum-free medium were seeded into upper chambers. The lower chambers were filled with 600 μl of RPMI 1640 containing 10% FBS as nutritional attractants. After 6 h of incubation, cells were fixed in 100% pre-cooling methanol for 30 min, and stained with crystal violet. Total cells were subjected to microscopic inspection. Five visual fields of each insert were randomly chosen under a light microscope.

### Colony formation assay

Twenty-four hours after transfection or treatment with miR-218 or miR-control,H1975 and A549 cells were treated with 0.25% trypsin plus 0.5 mM EDTA solution and re-plated in six-well plates at a density of 500 cells per well and were cultured with RPMI 1640 supplemented with 10% FBS for 10 days. At the end of the incubation period, the cells were washed twice with PBS, fixed in methanol, and dyed with crystal violet. Three independent experiments were performed.

### Bioinformatics analysis of miR-218 target genes

The biological targets of miRNA targets were predicted using the algorithms TargetScan, miRDB, PicTar and PITA [21–24].

### Luciferase assay

Double-stranded oligonucleotides corresponding to the wild-type (WT 3′-UTR) or mutant (Mut 3′-UTR) miR-218 binding site in the 3′-UTR of IL-6R and JAK3 genes were synthesized and inserted into the PmeI and XbaI sites of the pmirGLO Vector (Promega, Madison, WI), respectively.

The sequences of the wild-type and mutated IL-6R gene 3′-UTR used were 5′-AAACTAGCGGCCGCTAGT C*ATGGTTCT*G*TCAAG*CACCGCGT-3′ and 5′-AAACTAGCGGCCGCTAGT C*GCATCGTA*G*ATGTC*CACCGCT-3′, respectively (miRNA targeted and mutated bases are underlined).The sequences of the wild-type and mutated JAK3 gene 3′-UTR were 5′-AAACTAGCGGCCGCTAGTA*TGGTTCC*GTCAAGCACCATGG-3′ and 5′-AAACTAGCGGCCGCTAGTA*CATCGTA*GTCAAGCACCATGG-3′, respectively (miRNA targeted and mutated bases are underlined).

The wild-type or mutant luciferase reporter constructs, together with the pRL-TK Vector (Promega, Madison, WI), were co-transfected into cells with miR-218 mimic or mimic-control by lipofectamine 2000 (Life Technologies, Grand Island, NY).

Forty-eight hours after transfection, firefly and renilla luciferase activity was measured by the Dual-Luciferase Reporter Assay System (Promega, Madison, WI) according to the manufacturer’s protocol. Firefly luciferase activity was normalized to renilla luciferase activity. Three independent experiments were performed and the data is presented as mean ± SD.

### Real time PCR

Twenty-four hours after miR-218 mimic transfection, total RNA was isolated from H1975 and A549 cells using the miRNeasy Mini Kit (Qiagen, Valencia, CA) according to the manufacturer’s protocol. RNA concentrations were determined using the NanoDrop. Briefly, 500 ng of total RNA from each sample was subjected to reverse transcription using a High Capacity cDNA Reverse Transcription Kit (Applied Biosystems, Foster City, CA). Expression of the IL-6R or JAK3 gene was detected using RT^2^qPCR Primer Assays and RT^2^ SYBR Green Mastermixes (Qiagen, Valencia, CA) in an ABI 7900HT Sequence Detection System (Applied Biosystems, Foster City, CA). Relative gene expression was normalized to the expression of GAPDH and was calculated using the 2^(−ΔΔCT)^ method.

### Western blotting

Cells were lysed using the RIPA Lysis and Extraction Buffer (Life Technologies, Grand Island, NY) supplemented with protease inhibitors (Roche, Indianapolis, IN). The total protein was quantified with a Pierce BCA Protein Assay Kit (Pierce Biotechnology, Rockford, IL) according to the manufacturer’s protocol. Protein samples were separated by sodium dodecyl sulfate-polyacrylamide gel electrophoresis (SDS-PAGE) and transferred to a polyvinylidene fluoride (PVDF) membrane. After blocking in phosphate-buffered saline/Tween-20 containing 5% non-fat milk at room temperature for 1 h, the membrane was incubated with primary antibody (Cell Signaling Technology, Danvers, MA) at 4 °C overnight using human IL-6R antibody (1:2000, #12786), JAK3 antibody (1:2000, #8863), STAT3 antibody (1:2000, #4904), pSTAT3 antibody (1:1000, #9145), EGFR antibody (1:2000, #4267).Then, the membrane was incubated with goat anti-rabbit IgG secondary antibody conjugated with horseradish peroxidase (1:5000; Santa Cruz Biotechnology, Dallas, Texas). GAPDH (#2118) was used as a loading control. Proteins were visualized with LumiGLOchemiluminescent substrate (Cell Signaling Technology, Danvers, MA).

### Immunofluorescence

Forty-eight hours after miR-218 mimic transfection, cells were fixed with 50% methanol and 50% acetone at 4 °C for 30 min and blocked with 1% BSA for 1 h. After incubation with pSTAT3 or Ki67 primary antibody(Cell Signaling Technology, Danvers, MA), the cells were incubated with FITC or Alexa Fluor(R) 555-coupled secondary antibody(Cell Signaling Technology, Danvers, MA) for 1 h and then stained with the nuclear marker DAPI(Cell Signaling Technology, Danvers, MA) for 10 min. Fluorescence was observed with microscope.

### Aldefluor assay and FACS sorting

Aldefluor assay and Flow cytometry was used to quantify and enrich ALDH positive cancer cells with stem cell characterics since aldehyde dehydrogenase (ALDH) is a cancer stem cell associated marker in lung cancer [25, 26].The ALDH activity of H1975 and A549 was determined by using the Aldefluor assay kit (Stem Cell Technologies, Durham, NC). In brief, cells were dissociated into single cells by trypsin/EDTA digestion. Then, the single-cell suspension was washed twice in PBS without Ca^2+^/Mg^2+^and suspended in 1 ml Aldefluor assay buffer containing 5 μl ALDH substrate (1 ml/per 1 × 10^6^ cells) and incubated for 30–40 min at 37 °C in the dark. As a negative control, cells were treated with 5 μl of diethylaminobenzaldehyde (DEAB; 50 mmol/l), a specific ALDH inhibitor. For FACS sorting, cells were re-suspended in PBS buffer at 1 × 10^7^ cells per ml and run on an Aria cell sorter (BD Biosciences). The sorting gates were established, by negative control cells which were treated with the ALDH inhibitor DEAB.

### Tumorsphere culture

After FACS sorting, the ALDH^+^ H1975 cells and ALDH^+^ A549 cells were placed in ultralow attachment 6-well plates (Corning, NY)at a density of 2000 cells per well in serum-free stem cell medium (MEBM). Twenty-four hours after seeding, the ALDH1A1 positive cells were infected with lentivirus expressing miR-218 or miR-control. Every three to four days, half of the medium was replaced. After 10–14 days, spheroid formation was checked and representative images were taken.

### Mouse xenograft tumor model

All the animal studies were conducted according to protocols approved by the Ethical Committee of Hua Zhong University of Science and Technology, People’s Republic of China. And all the animals were kept according to the Animal Care Guidelines and housed in 12 h light/dark conditions with free access to food and water. Human lung cells A549 were infected with lentivirus expressing miR-218 or miR-control. Forty-eight hours after infection, cells were harvested and were counted by Handheld Automated Cell Counter Scepter™ (Millipore, Bilerica, MA). 5 × 10^6^ cells were subcutaneous injected into 6 weeks old nude mice (SJA Lab Animal, Hunan, China). Tumor growth was monitored once a week by caliper measurements (LxWxD). At fifth week after injection, mice were sacrificed and tumors were collected for analysis.

### Statistical analysis

Differences between experimental groups and controls were assessed by Student’s t-test. Two-tailed tests were used, and a *P*-value of 0.05 or less was considered statistically significant. Data is shown as mean ± SD of three independent experiments.

## Results

### Expression levels of miR-218 and its host gene *SLIT2/3* in normal and lung tumor tissues

**Fig. 1:**
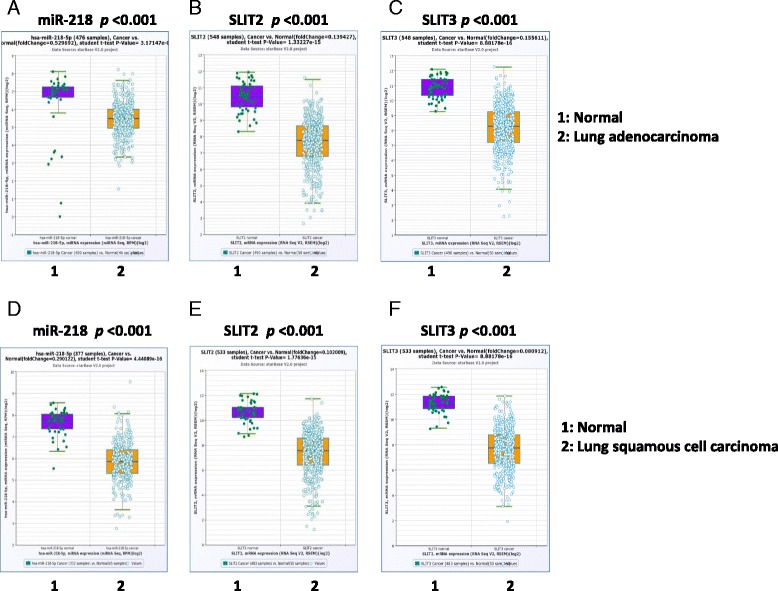
MiR-218 correlates with tumorigenesis in lung cancer. **a** The expression levels of miR-218 in 430 lung adenocarcinoma tumor tissues and 46 normal tissues were obtained from TCGA. **b**-**c** The expression levels of SLIT2 and SLIT3 in 490 lung adenocarcinoma tumor tissues and 58 normal tissues were obtained from TCGA. (**d**) The expression levels of miR-218 in 332 lung squamous cell carcinoma tumor tissues and 45 normal tissues were obtained from TCGA. **e**-**f** The expression levels of SLIT2 and SLIT3 in 483 lung squamous cell carcinoma tumor tissues and 50 normal tissues were obtained from TCGA

The levels of miR-218 were found to be significantly lower in lung adenocarcinoma tissues and were compared to normal lung tissues (fold change of cancer vs. normal = 0.53, *p* = 3.2 × 10^−8^) (Fig. 1a) using the existing data from TCGA and following the relevant algorithms (http://starbase.sysu.edu.cn) [27, 28]. To further investigate lung tumorigenesis, we also analyzed the expression of *SLIT2* and *SLIT3* since miR-218 co-expresses with these host tumor suppressor genes. The data revealed that the levels of *SLIT2* and *SLIT3 were also* significantly lower with fold change of *SLIT2* to be 0.14 (*p* = 1.3 × 10^−15^) in lung adenocarcinoma tissues when compared to normal lung tissues (Fig. 1b). Similarly, the fold change of *SLIT3* in cancer vs. normal tissues was 0.16 (*p* = 8.9 × 10^−16^) (Fig. 1c).

Additionally, we also compared the levels of miR-218, *SLIT2* and *SLIT3* in lung squamous cell carcinoma subtype and normal tissues. Similar low expression levels of miR-218, *SLIT2*and *SLIT3* were obtained in lung cancer tissues when compared to normal lung tissues (the fold change of miR-218 in cancer vs. normal = 0.29, *p* = 4.4 × 10^−16^, Fig. 1d; the fold change of *SLIT2* in cancer vs. normal = 0.10, *p* = 1.8 × 10^−15^, Fig. 1e; the fold change of *SLIT3* in cancer vs. normal = 0.08, *p* = 8.9 × 10^−16^, Fig. 1f). These data suggest that both miR-218 and the related host genes were downregulated in both of lung cancer NSCLC subtypes.

### Overexpression of miR-218 affects lung cancer cell proliferation and invasiveness

**Fig. 2:**
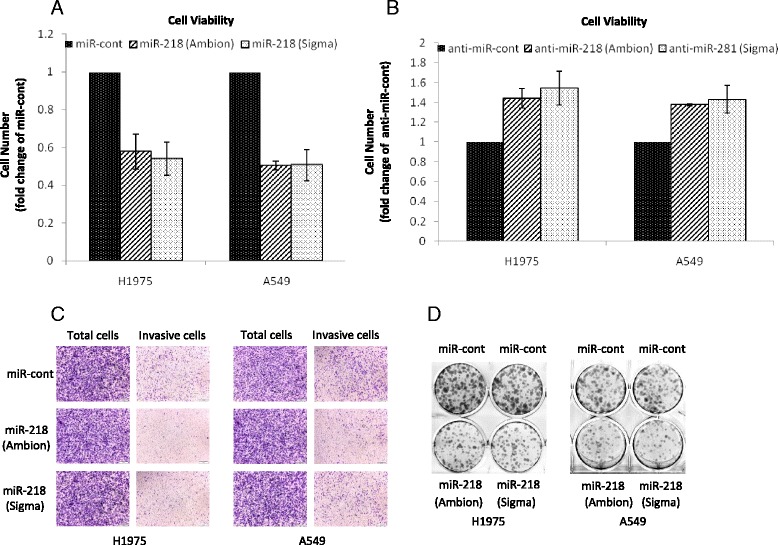
Functional studies of miR-218 in lung cancer cells. **a** Cell proliferation effects in H1975 and A549 cells were determined 48 h after transfection of miR-218 or miR-control. **b** Cell proliferation was determined in H1975 and A549 cells 48 h after transfection of anti-miR-218 or anti-miR-control. **c** Trans-well invasion assay using H1975 and A549 cells transfected with miR-218 or miR-control. **d** Colony formation capability in H1975 and A549 cells was examined after transfection of miR-218 or miR-control. The data were the means ± SD of three individual experiments

To study whether re-expression of miR-218 would affect cell behavior, we transfected miR-218 or anti-miR-218 into A549 and H1975 cells and then performed cell proliferation, trans-well invasion, and colony formation assays. The results of cell proliferation showed that re-expression of miR-218 reduced cell proliferation (Fig. 2a), whereas overexpression of anti-miR-218 promoted cell proliferation (Fig. 2b). Our data also demonstrated that overexpression of miR-218 decreased cell invasion (Fig. 2c) and colony formation ability (Fig. 2d).

### MiR-218 negatively regulates IL-6/JAK/STAT3 pathway

**Fig. 3:**
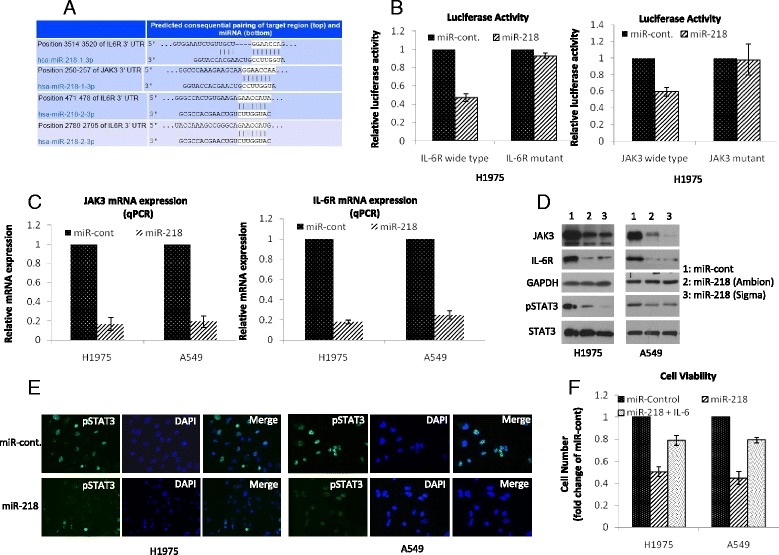
MiR-218 directly targets IL-6R and JAK3 in lung cancer cells. **a** IL-6R and JAK3 gene was predicted as a direct target of miR-218 (TargetScan). **b** Relative luciferase activity was measured after luciferase reporter plasmids with IL-6R or JAK3 3′-UTR constructs (either wild-type or mutant) or control reporter plasmid were co-transfected into H1795 cells with miR-218 or miR-control, respectively. The data were the means ± SD of three individual experiments. **c** The mRNA expression of IL-6R and JAK3 was analyzed by quantitative real-time PCR 24 h after transfection of miR-218 or miR-control. **d** Protein levels of JAK3, IL-6R, pSTAT3 and STAT3 in A549 and H1795 cells were examined by western blotting 36 h after transfection of miR-218 or miR-control. **e** The phosphorylated STAT3 in H1975 and A549 cells was determined by immunofluorescence 36 h after transfection of miR-218 or miR-control. **f** Cell proliferation was determined by cell counting using H1975 and A549 cells 48 h after transfection of miR-218 or miR-control with or without IL-6 treatment

**Fig. 4:**
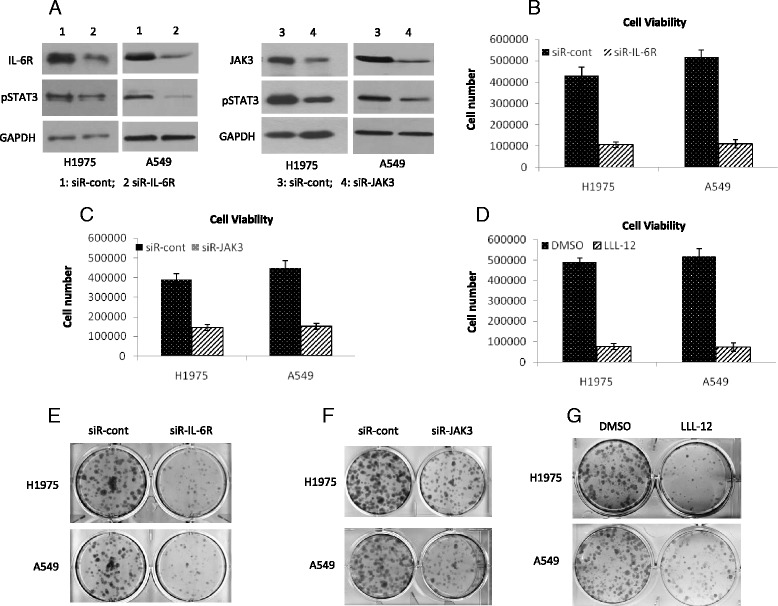
Inhibition of IL-6/JAK3/STAT3 signaling reduces cell proliferation and colony formation in vitro. **a** A549 and H1795 cells were transfected with siR-IL-6R, or siR-JAK3. Twenty-four hours after transfection, cells were lysed and IL-6R, JAK3, pSTAT3 and GAPHD were analyzed by western blotting. **b**-**d** A549 and H1795 cells were transfected with siR-IL-6R (**b**) or siR-JAK3 (**c**) or were treated with 1 μL of LLL-12 (**d**). Seventy-two hours after transfection or treatment, cells were counted. The data shown were the means ± SD of three individual experiments. e-g A549 and H1795 cells were transfected with siR-IL-6R (**e**) or siR-JAK3 (**f**) or were treated with 1 μL of LLL-12 (**g**). Ten days after transfection or treatment, cells were fixed and stained by crystal violet

To further gain insights by which miR-218 could potentially regulate the cell growth and differentiation of lung cancer cells, we performed predictions using programs TargetScan, miRDB, PicTar and PITA. It was predicted that miR-218 could target components of IL-6/JAK/STAT3 pathway. TargetScan showed that both IL-6R and JAK3 had a score of 94, respectively (Fig. 3a). Therefore, it could be asserted that miR-218 could portray its inhibitory effects by at least partially modifying IL-6/JAK/STAT3 signaling mediator molecules. To determine whether the IL-6R and JAK3 mRNA expression is regulated by miR-218 through direct binding to their 3′-UTR regions, we used a dual-luciferase reporter system containing either the wild-type or the mutated 3′-UTR of IL-6R and JAK3. The relative luciferase activity decreased in H1795 cells transfected with luciferase reporter pmirGLO-3′ UTR-wild-type and miR-218 compared with the negative control miR-cont. This suppressive effect was abolished by the mutations in the miR-218 targeted 3′ UTR regions (Fig. 3b). To further gain evidence, we examined the mRNA and protein expression of IL-6/JAK/STAT3 pathway after overexpressing miR-218 by qRT-PCR and western blotting. Overexpression of miR-218 significantly reduced the expression of JAK3, IL-6R and phosphorylated STAT3 (Fig. 3c, d). Furthermore, immunofluorescence staining revealed that overexpression of miR-218 decreased phosphorylation of STAT3 (Fig. 3e). To validate these findings we examined whether activating STAT3 signal with IL-6 treatment can rescue cells upon miR-218 transfection, we treated miR-218 transfected cells with IL-6. Our data showed that IL-6 treatment partially rescued cells transfected by miR-218 (Fig. 3f). Taken together, these data suggest that miR-218 modulated lung cancer cell phenotype through negatively regulating the STAT3 signaling pathway. Additionally, we also investigated the effect of STAT3 pathway inhibition in lung cancer cells to understand the importance of IL-6/JAK/STAT3 pathway. We decreased IL-6R and JAK3 with siRNA or decreased phosphorylated STAT3 with a STAT3 inhibitor, LLL12 [29, 30].Decreasing either IL-6R or JAK3 reduced the levels of phosphorylated STAT3 (Fig. 4a). Cell based assays demonstrated that treating cells with siR-IL-6R, siR-JAK3 or LLL12 inhibited cell proliferation (Fig. 4b-d) and colony formation (Fig. 4e-g). These results confirmed the establishment of IL-6/STAT3 signaling in currently studied lung cancer cells. In addition to IL-6R and JAK3, miR-218 was also reported to negatively regulate EGFR, leading to reduced levels of pSTAT3 [31]. We confirmed the previous results (Additional file 1: Figure S1A) and found downregulation of EGFR decreased the levels of pSTAT3 more significantly in EGFR mutated cells (H1975) than in EGFR wild type cells (A549) (Additional file 1: Figure S1B). Similarly, downregulating of EGFR reduced cell viability more significantly in EGFR mutated cells (H1975) than in EGFR wild type cells (A549) (Additional file 1: Figure S1C). Overall, our data indicated that miR-218 negatively regulated STAT3 signaling through IL-6R and JAK3 in EGFR wild type cells and through IL-6R, JAK3, and EGFR in EGFR mutated cells.

### MiR-218 is downregulated in ALDH positive lung cancer cells

**Fig. 5:**
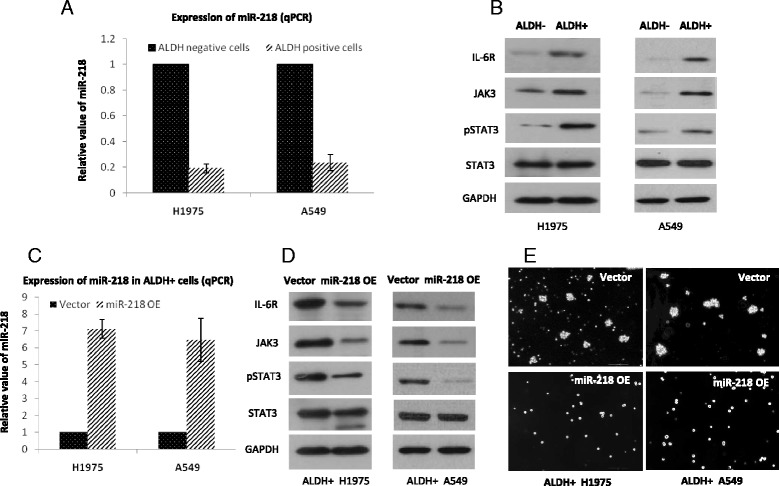
MiR-218 regulates the survival of ALDH positive lung cancer cells through STAT3 signaling. **a** ALDH1A1 positive cells were isolated from H1975 and A549 cells and the levels of miR-218 were measured by real time PCR. **b** The levels of IL-6R, JAK3 and pSTAT3 in ALDH1A1 positive cells were analyzed by western blotting. **c** ALDH1A1 positive cells were infected by lentivirus expressing miR-218 or miR-control. The levels of miR-218 in these infected cells were examined by real time PCR. **d** The levels of IL-6R, JAK3, phosphorylated STAT3, STAT3 and GAPDH in the infected cells were determined by western blotting. **e** The ALDH1A1 positive cells were plated as single cells in ultra-low attachment six-well plates and were cultured in a serum-free MEBM. Twenty-four hours after seeding, the ALDH1A1 positive cells were infected with lentivirus expressing miR-218 or miR-control

ALDH positive lung cancer cells have shown some of the characterics of cancer stem cells, such as drug resistance [26]. To explore whether miR-218 would be dysregulated in these small populations of cells marked by ALDH1A1, we isolated ALDH1A1 positive cells from both H1975 and A549 cells by flow cytometry. We compared the miR-218 levels in ALDH1A1 positive and ALDH1A1 negative cells by Real Time PCR and found that ALDH1A1 positive cells had lower levels of miR-218 (Fig. 5a). We further observed that ALDH1A1 positive cells had higher levels of IL-6R, JAK3 and phosphorylated STAT3 (Fig. 5b), suggesting that the lower levels of miR-218 may be responsible for the upregulation of STAT3 signaling in ALDH positive lung cancer cells. We overexpressed miR-218 in ALDH1A1 positive cells by lentivirus expressing miR-218 (Fig. 5c) and found that the levels of IL-6R, JAK3 and phosphorylated STAT3 were reduced (Fig. 5d). Additionally, overexpression of miR-218 inhibited ALDH1A1 positive to survive in anchorage-independent conditions and their ability to form tumor-spheres (Fig. 5e).

### Overexpression of miR-218 reduces tumor growth in vivo by targeting STAT3 signaling

**Fig. 6:**
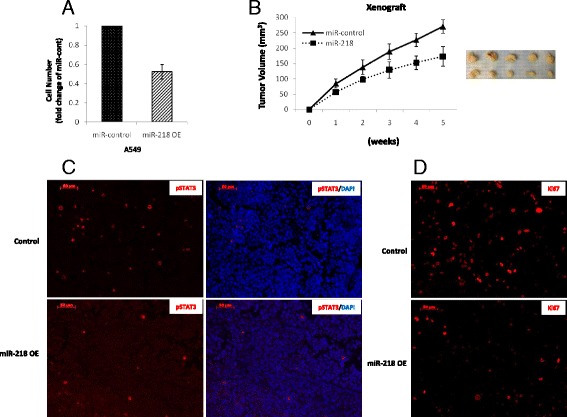
Overexpression of miR-218 represses tumor growth in vivo. **a** A549 cells were infected with lentivirus expressing miR-218 or miR-control. Forty-eight hours after infection, cell proliferation was determined. **b** 5 × 10^6^ A549 cells infected with lentivirus expressing miR-218 or miR-control were injected subcutaneously into two groups of nude mice (five mice per group). Tumor volumes were measured once per week. **c** and **d** Tissue levels of phosphorylated STAT3 and Ki67 in an A549 xenograft were visualized by immunofluorescence co-staining with DAPI

To investigate whether overexpression of miR-218 would reduce tumor growth in vivo, we infected A549 cells with lentivirus expressing miR-218. Cells infected with miR-218 expressing virus showed reduced cell proliferation compared to cells infected with control virus (Fig. 6a). Further evidence was acquired by injecting these infected cells subcutaneously into nude mice. Compared to the control group, the mean volume of the tumors in the miR-218 overexpressing group was significantly smaller (Fig. 6b). Subsequently, we assessed STAT3 signaling and proliferation via staining for phosphorylated STAT3 (Fig. 6c) and Ki-67 (Fig. 6d). Compared with the tumors from the control group, tumor tissues with miR-218 overexpression showed lower levels of phosphorylated STAT3(Fig. 6c) and Ki67 (Fig. 6d), suggesting that overexpressing miR-218 inhibited the STAT3 signaling and reduced tumor growth in vivo.

### MiR-218 host genes and the molecules of IL-6/STAT3 signaling pathway correlate with prognosis of lung cancer patients

**Fig. 7:**
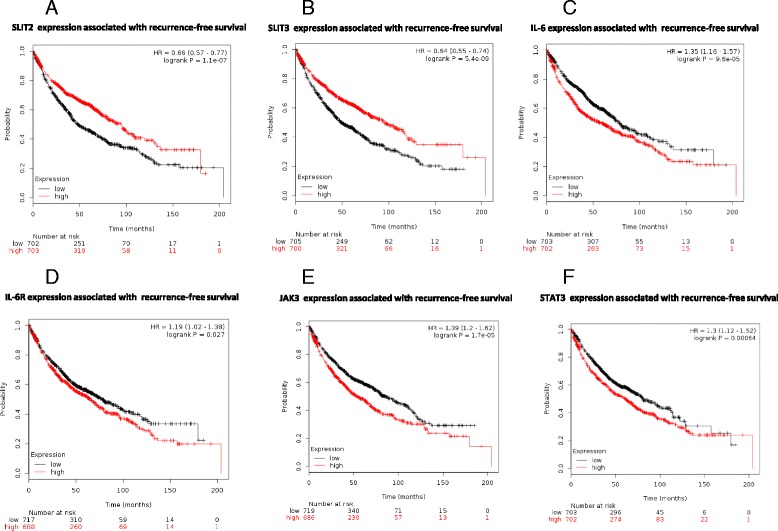
The components of the IL-6/JAK/STAT3 pathway and miR-218 host genes correlate with prognosis in lung cancer patients. **a** Kaplan–Meier curves for recurrence free survival were created using the Kaplan–Meier Plotter (www.kmplot.com) with lung cancer patients classified according to high and low SLIT2 (**a**), SLIT3 (**b**), IL-6 (**c**), IL-6R (**d**), JAK3 (**e**) and STAT3 (**f**) gene expression. Hazard ratio (with 95% confidence interval) and log-rank *p* values were calculated

In order to correlate our findings with lung cancer prognosis in clinical settings, we analyzed the data from 1405 lung cancer patients and the gene expression of miR-218 host genes (*SLIT2/SLIT3*), *IL-6*, *IL-6R*, *JAK3* and *STAT3* in their tumor tissues [32]. Median levels of gene expression were used for all six genes as cutoff points. Both miR-218 and host genes, *SLIT2* and *SLIT3* were negatively correlated with poor prognosis (Fig. 7a, b), whereas *IL-6*, *IL-6R*, *JAK3* and *STAT3* were positively correlated with poor prognosis (Fig. 7c-f). We also analyzed the correlation between levels of IL6-R and miR-218, as well as the correlation between levels of JAK3 and miR-218 in patients with lung adenocarcinima TCGA cohort, which showed significant negative correlation (Additional file 1: Figure S2).

## Discussion

In normal cell physiology, miRNAs regulate the precision of gene expression to modulate cell signaling pathways for regular growth and differentiation [33]. However, cancer cells display dysregulation of miRNA expression through different mechanisms, including abnormal transcription, epigenetic changes, amplification or deletion of miRNA host genes [34]. The advances in this field have brought new diagnostic and therapeutic opportunities for patients affected with different types of cancer. Clinical trials using miRNAs started in 2013 and studies have been conducted in breast cancer, liver cancer, glioma, and non-small-cell lung cancer towards identifying miRNAs as biomarkers. In such patients new opportunities towards molecular diagnostics and therapies could emerge when investigating responses of miRNAs and underlying molecular mechanisms behind cancer progression [35]. In this study, we evaluated the diverse roles of miR-218 in human lung cancer, investigated the molecular mechanisms, and determined whether it could serve as a biomarker of prognosis providing newer insights towards therapeutics.

Lung cancer is the most leading cancer death in men all over the world, especially in China [1, 36]. Despite recent advances in the diagnostics and treatment, the prognosis of patients with lung cancer remains poor. For example, almost all NSCLC patients while initially responding to EGFR inhibitor treatment eventually develop disease resistance [37]. Studies involved in evaluating the role of miRNAs showed dysregulation of miRNA expression in NSCLC and postulated its important role toward malignancy [38]. These miRNAs serve as either oncogenes or tumor suppressors. It is seen that the expression of miR-17, miR-18a, miR-19, miR-20a, miR-21, miR-31, miR-92a, and miR-224 is upregulated in lung cancer cells and inhibition of their expression can reduce cell growth and invasion capacities [7, 39–41]. Others, such as let-7, miR-34, and miR-126 are downregulated in human lung cancer and their overexpression is implicated in reducing cellular growth and invasion capacities [42–44]. A previous study highlighted that the levels of miR-218 is downregulated in the tissues of lung squamous cell carcinoma, in association with cigarette smoking [17]. It should be noted that several factors other than cigarette smoking are responsible factors towards development of NSCLC. Our study using lung cancer samples included in TCGA database reaffirmed the results of the previous study in lung squamous cell carcinoma and also found that the levels of miR-218 and its host genes *SLIT2/3* are downregulated in the tissues of lung adenocarcinoma. Overexpression of miR-218 in lung cancer cells reduced cell proliferation and invasiveness, suggesting miR-218 is acting as tumor suppressor consistent with its function in other reported human cancer types [16, 18–20].

MiR-218 has been reported to target EGFR [31], an important kinase implicated in signaling in lung cancer cells. We found that miR-218 also targeted two molecules of IL-6/JAK/STAT3 signaling pathway, which is constitutively activated in NSCLC [45]. Abnormalities in the IL-6/JAK/STAT3 pathway are also due to expression of a variety of oncogenes, such as Myc and VEGF, reportedly increased in lung cancer and other cancer types [46, 47]. Persistent activation of STAT3 can be caused by either the reduced levels of negative regulators, such as PIAS, or the increased levels of kinases or receptors [45]. Our data revealed a novel mechanism, by which STAT3 is persistently activated in lung cancer. MiR-218 was downregulated in NSCLC, which then caused constitutive activation of STAT3 signaling. Overexpressing miR-218 in lung cancer reduced STAT3 signaling when tested both in vitro and in vivo. In addition, in EGFR mutated lung cancer cells, miR-218 can also negatively regulate pSTAT3 signaling through targeting EGFR [31].

More importantly, we found that ALDH positive lung cancer cells, marked by ALDH1A1, had lower levels of miR-218 and higher levels of STAT3 signaling than in ALDH negative cells. This small population may be responsible for tumor maintenance. These cells are hypothesized to persist in tumors as a distinct population and cause relapse and metastasis by giving rise to new tumors [48]. Therefore, development of specific therapies targeting these cells holds hope for improvement of survival and quality of life of cancer patients, especially for patients with metastatic disease.The dysregulation of miRNAs have been implicated in regulating cancer stem cells or drug resistant cells [49].Therefore, a better understanding of miRNA expression in cancer stem cells or drug resistant cells and the molecular mechanisms may help identify new therapeutic targets. Our findings that miR-218 was downregulated in ALDH positive lung cells aid to clarify the mechanism of upregulation of STAT3 signaling in these cells. It has been shown that targeting JAK/STAT3 with inhibitors results in downregulating self-renewal capability of lung cancer stem cells and displays reduced resistance to multiple cancer drugs [50]. Our data suggest that overexpressing miR-218 in ALDH positive lung cancer cells could also exhibit similar responses in reducing self-renewal capability of these cells.

In a previous study, miR-218 was reported to positively regulate STAT3 in the spinal cord and microglia through targeting SOCS3 [51]. However, SOCS3, a tumor suppressor, is silenced in NSCLC [52, 53]. Therefore, miR-218 is not able to positively regulate STAT3 in NSCLC, whose SOCS3 is silenced.

In summary, miR-218 is capable to inhibit lung cancer cell proliferation and invasion, at least partially through repressing IL-6R and JAK3 genes expression. The newly-identified miR-218–mediated IL-6R and JAK3 genes silencing may facilitate a better understanding of the molecular mechanisms of lung cancer progression and present a new strategy to treat patients with lung cancer.

## Conclusions

Overall, our findings of the anti-proliferative effects of miR-218 in lung adenocarcinima cells, through the direct targeting of IL-6R and JAK3, further corroborate a tumor suppressive role for miR-218 in NSCLC. Especially considering the significant decrease of miR-218 in ALDH positive cells with cancer stem cell characterics, further investigation of IL-6/STAT3 as a potentially therapeutic target in NSCLC is warranted.

## Additional file

Additional file 1: Figure S1. (A) A549 and H1975 cells were transfected with miR-cont or miR-218. Protein levels of EGFR were examined by western blotting 36 h after transfection. (B) A549 and H1975 cells were transfected with siR-cont or siR-EGFR. Protein levels of EGFR and pSTAT3 were examined by western blotting 36 h after transfection. (C) Cell proliferation in H1975 and A549 cells was determined 48 h after transfection of siR-EGFR or siR-control. **Figure S2.** Correlation of IL-6R/JAK3 and miR-218 in patients with lung adenocarcinoma in TCGA cohort. (PPT 4631 kb)

## Abbreviations

ALDH: Aldehyde dehydrogenase
IL-6R: Interleukin-6 receptor
JAK/STAT3: Janus kinase /signal transducer and activator of transcription 3
MiR-218: microRNA-218
NSCLC: Non-small-cell lung carcinomas
TCGA: The Cancer Genome Atlas
