# Supplementary material for: MicroRNA-218 functions as a tumor suppressor in lung cancer by targeting IL-6/STAT3 and negatively correlates with poor prognosis
Source: Mol Cancer. 2017 Aug 22;16:141. doi: 10.1186/s12943-017-0710-z (PMC5567631; doi:10.1186/s12943-017-0710-z)
Supplement: Additional file 1: Figure S1. — (A) A549 and H1975 cells were transfected with miR-cont or miR-218. Protein levels of EGFR were examined by western blotting 36 h after transfection. (B) A549 and H1975 cells were transfected with siR-cont or siR-EGFR. Protein levels of EGFR and pSTAT3 were examined by western blotting 36 h after transfection. (C) Cell proliferation in H1975 and A549 cells was determined 48 h after transfection of siR-EGFR or siR-control. Figure S2. Correlation of IL-6R/JAK3 and miR-218 in patients with lung adenocarcinoma in TCGA cohort. (PPT 4631 kb) [file 12943_2017_710_MOESM1_ESM.ppt]

## Slide 1
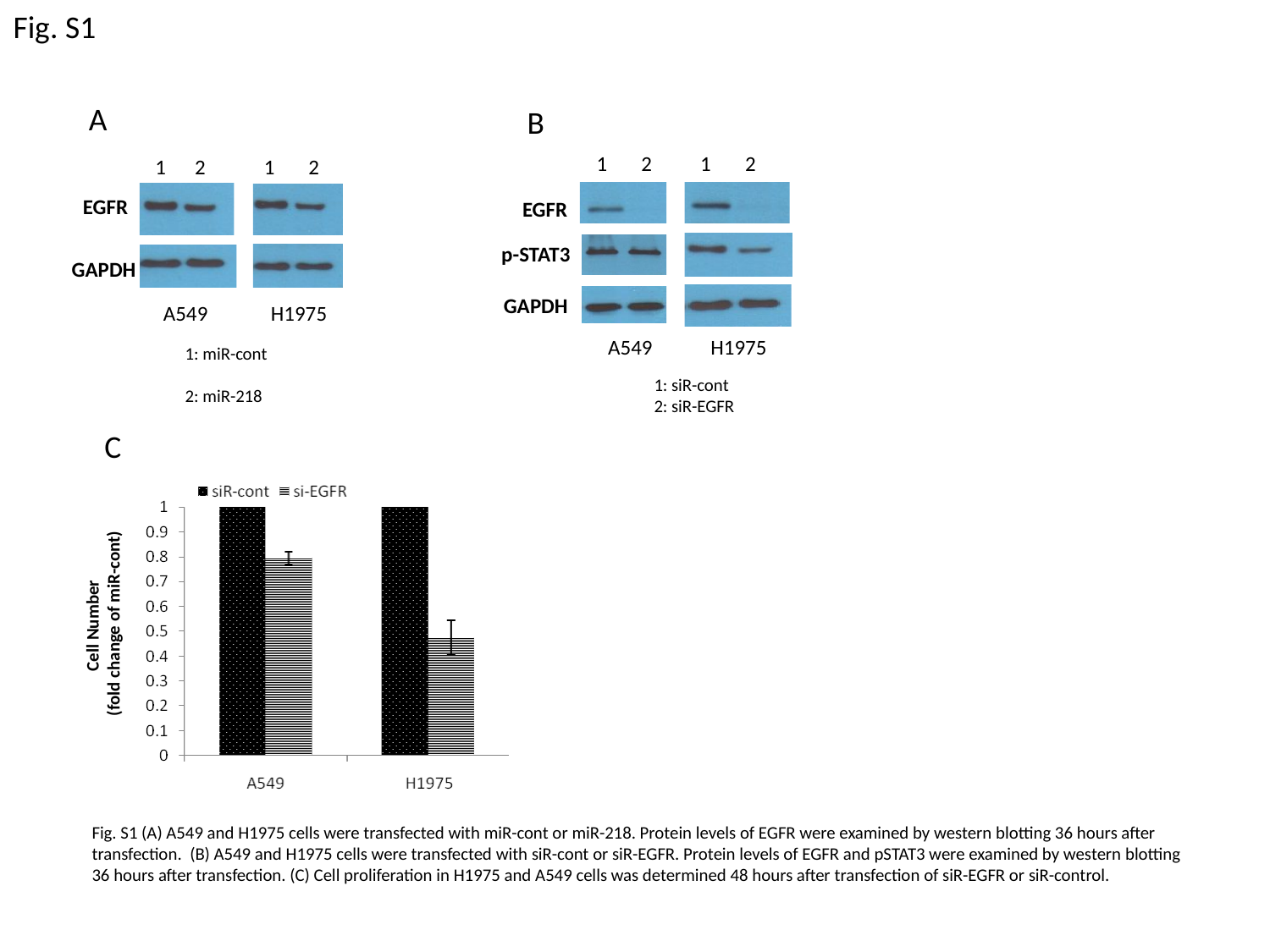

Fig. S1
A
B
1 2 1 2
EGFR
p-STAT3
GAPDH
A549 H1975
1: siR-cont
2: siR-EGFR
1 2 1 2
EGFR
GAPDH
A549 H1975
1: miR-cont
2: miR-218
C
Cell Number
(fold change of miR-cont)
Fig. S1 (A) A549 and H1975 cells were transfected with miR-cont or miR-218. Protein levels of EGFR were examined by western blotting 36 hours after transfection. (B) A549 and H1975 cells were transfected with siR-cont or siR-EGFR. Protein levels of EGFR and pSTAT3 were examined by western blotting 36 hours after transfection. (C) Cell proliferation in H1975 and A549 cells was determined 48 hours after transfection of siR-EGFR or siR-control.

## Slide 2
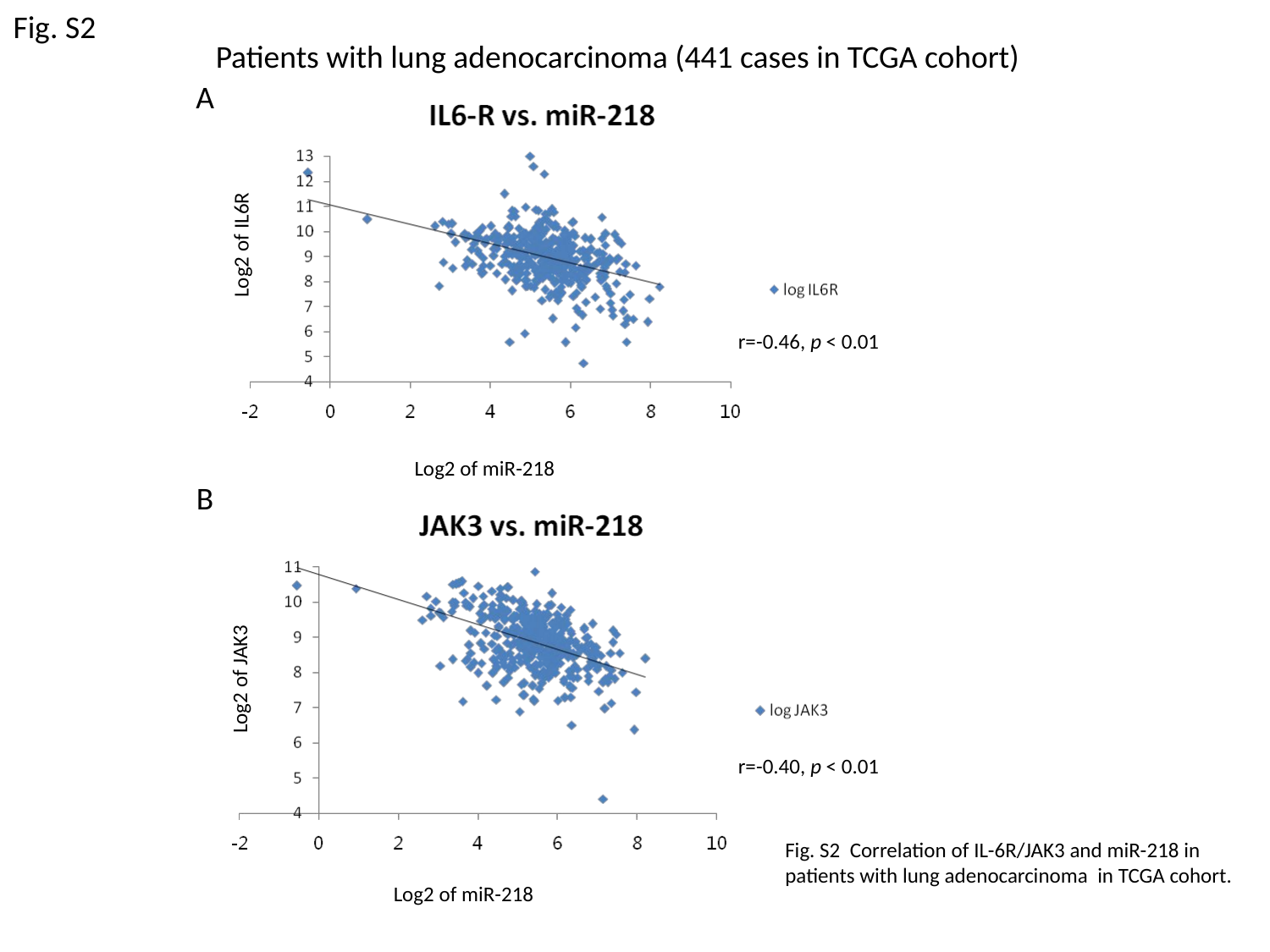

Fig. S2
Patients with lung adenocarcinoma (441 cases in TCGA cohort)
A
Log2 of IL6R
r=-0.46, p < 0.01
Log2 of miR-218
B
Log2 of JAK3
r=-0.40, p < 0.01
Fig. S2 Correlation of IL-6R/JAK3 and miR-218 in patients with lung adenocarcinoma in TCGA cohort.
Log2 of miR-218
